# Intracellular Calcium Determines the Adipogenic Differentiation Potential of Human Umbilical Cord Blood-Derived Mesenchymal Stem Cells via the Wnt5a/*β*-Catenin Signaling Pathway

**DOI:** 10.1155/2018/6545071

**Published:** 2018-07-11

**Authors:** Yun Kyung Bae, Ji Hye Kwon, Miyeon Kim, Gee-Hye Kim, Soo Jin Choi, Wonil Oh, Yoon Sun Yang, Hye Jin Jin, Hong Bae Jeon

**Affiliations:** Biomedical Research Institute, MEDIPOST Co. Ltd., Seongnam 13494, Republic of Korea

## Abstract

Mesenchymal stem cells- (MSCs-) based therapies show different degrees of efficacies for the treatment of various diseases, including lipogenesis. We evaluated the adipogenic differentiation ability of human umbilical cord blood-derived MSCs (hUCB-MSCs) from different donors and examined the contribution of the intracellular calcium (Ca^2+^) level to this diversity. hUCB-MSCs treated with Ca^2+^ or the Ca^2+^ chelator BAPTA-AM increased and decreased adipogenic differentiation, respectively. Canonical Wnt5a/*β*-catenin expression decreased during adipogenic differentiation of hUCB-MSCs. Treatment with Wnt5a blocked the adipogenic differentiation of hUCB-MSCs and activated the Wnt pathway, with a decrease in the adipogenesis markers PPAR*γ* and leptin, and reduced lipid vacuole-associated Oil red O activity. In contrast, inhibition of the Wnt pathway with dickkopf-1 and *β*-catenin small interfering RNA transfection promoted the adipogenic potential of hUCB-MSCs. Interestingly, the Ca^2+^-based system exhibited a synergic effect on adipogenic potential through the Wnt5a/*β*-catenin pathway. Our data suggest that the variable adipogenic differentiation potential of hUCB-MSCs from different lots is due to variation in the intracellular Ca^2+^ level, which can be used as a marker to predict hUCB-MSCs selection for lipogenesis therapy. Overall, these results demonstrate that exogenous calcium treatment enhanced the adipogenic differentiation of hUCB-MSCs via negatively regulating the Wnt5a/*β*-catenin signaling pathway.

## 1. Introduction

Large soft tissue defects due to an external wound or surgical resection are very common in clinical practice, and adipose tissue regeneration is a very important part of reconstruction plastic surgery that allows for the recovery of such tissue defects. Thus, the demand for soft tissue reconstruction for the regeneration of damaged tissues or plastic surgery is rapidly increasing. Adipogenesis can be exploited or enhanced in various fields related to soft tissue reconstruction. Recently, various transplant methods using stem cells have been developed for the purpose of regenerating tissues damaged due to burn injury, surgical resection, innate defects, breast tissue reconstruction, and so forth [1–4].

In particular, mesenchymal stem cells (MSCs) are multipotent cells that can differentiate into various cell types such as adipocytes, osteocytes, chondrocytes, and muscle cells. MSCs have various advantages, including the abilities for tissue regeneration and immunoregulation, and they are easily isolated from various tissues such as the bone marrow (BM), adipose tissue (AT), umbilical cord (UC), and umbilical cord blood (UCB) [5–7]. Among these, human umbilical cord blood-derived MSCs (hUCB-MSCs) are easily collected and have advantages such as a high proliferation rate, expression of various beneficial trophic factors, and low immunological rejection during allogeneic transplantation due to their low immunogenicity and excellent immunoregulation capacity [8–10]. Thus, hUCB-MSCs have a wide range of clinical applications and are considered an important source for stem cell therapy development.

However, in some studies, hUCB-MSCs applied to a damaged area that includes the adipose tissue showed poor results for regeneration. For example, hUCB-MSCs were injected in a mouse injury model with scars created through a subcutaneous route, but no regeneration in the damaged area was observed [11]. By contrast, regeneration at the scar site occurred well after AT-MSCs or AT-MSCs that had gone through adipogenic differentiation were transplanted to the scar area of a clinical patient [12, 13]. Based on these reports, it is currently considered that the adipogenic differentiation potential of hUCB-MSCs is not sufficient in some cases due to donor-to-donor variability. Indeed, there is heterogeneity in several aspects of the UCB itself, which then affects the cell size, proliferation capacity, and differentiation potential of the hUCB-MSCs isolated from various donors [14–16]. Among this heterogeneity, the adipogenic differentiation potential shows the greatest degree of variation among different donors [17], which is currently the main limitation for the clinical application of these cells with other various advantages in adipose tissue regeneration.

Calcium performs the physiological functions of cells by activating various intracellular signaling pathways [18, 19]. Similarly, calcium plays an important role in the cell proliferation and differentiation of MSCs [20]. In particular, calcium is known to accelerate the early stage of adipocyte differentiation through cAMP induction and regulates the expression of various genes involved in adipogenic differentiation through peroxisome proliferator-activated receptor gamma (PPAR*γ*) [21, 22]. Furthermore, Wnt, a glycoprotein that has many cysteine residues and is secreted from the cell, regulates the level of *β*-catenin, which is a key effector molecule that is involved in regulating the expression of various target genes [23, 24]. In particular, many studies have reported that Wnt/*β*-catenin signaling is related to the differentiation or maintenance of the self-renewal of stem cells [25, 26]. In fact, MSCs express various Wnt ligands (e.g., Wnt2, Wnt3, Wnt4, and Wnt10), receptors, and inhibitors [27, 28]. Several studies have reported that canonical Wnt signaling (Wnt3 and Wnt10) activates the differentiation of preadipocytes into adipocytes through multiple pathways, including those involving *β*-catenin [29, 30]. However, little is known about the mechanism of adipogenic differentiation of hUCB-MSCs, especially with respect to the relationship between calcium and the Wnt/*β*-catenin pathway.

Therefore, the objective of the present study was to investigate the molecular mechanism and variation in the differentiation potential of hUCB-MSCs from different individual donors. Toward this end, we investigated the association between the intracellular calcium level and adipogenic differentiation potential of hUCB-MSCs from different individual donors and confirmed the role of Wnt/*β*-catenin signaling and specific regulators in adipogenic differentiation.

## 2. Methods

### 2.1. Cell Preparation

This study was approved by the Institutional Review Board of MEDIPOST Co. Ltd. (MP-2016-07-1). hUCB was collected from umbilical veins after neonatal delivery after obtaining informed maternal consent. hUCB harvests were processed within 24 h of collection. The hUCB was isolated by separating mononuclear cells (MNCs) with Ficoll-Hypaque solution (*d* = 1.077 g/cm^3^; Sigma-Aldrich, St. Louis, MO, USA). The separated MNCs were washed and suspended in minimum essential medium alpha (Gibco/Invitrogen, Carlsbad, Grand Island, NY, USA) supplemented with 10% fetal bovine serum (FBS; Gibco). Cultures were maintained at 37°C in a humidified atmosphere containing 5% CO_2_, wherein the culture medium was changed twice a week [31]. The expansion of live cells was analyzed using the trypan blue exclusion method. For expansion, MSCs were cultured for 5 days, harvested with trypsin-ethylenediaminetetraacetic acid (Gibco), counted, and then reseeded at a cell density of 2000 cells/cm^2^. In this study, we used six hUCB-MSC lines that were isolated from UCB samples obtained from different donors; the basic information of these samples is summarized in Supplementary Table 1. Both BM-MSCs and AT-MSCs were purchased from Cambrex (Walkerville, MD, USA). The calcium chloride solution was purchased from Sigma-Aldrich. The intracellular calcium chelator BAPTA-AM was purchased from Calbiochem (La Jolla, CA, USA). Wnt5a and dickkopf-1 (Dkk-1) were obtained from R&D System.

### 2.2. Calcium Measurement

Rhod2-AM (excitation wavelength, 552 nm; emission wavelength, 581 nm) and Fluo4-AM (excitation wavelength, 494 nm; emission wavelength, 506 nm) were used to measure the intracellular calcium level. The fluorescent dye Rhod2-AM ester (Life Technologies, USA) of the probe is cell-permeant and rapidly cleaves in the mitochondria to yield the Rhod2 indicator, which displays a large increase in fluorescence intensity upon binding to Ca^2+^ [32]. Fluo4-AM (Molecular Probes, Eugene, OR, USA) is a dye molecule that fluoresces only when bound to calcium; this reaction allows for the release of free cytoplasmic calcium to be visualized utilizing fluorescence by flow cytometry or microscopy [33]. For flow cytometry, the cells were harvested, pelleted, and suspended in phosphate-buffered saline (PBS; Gibco) containing 10% FBS. Calcium levels with treatment of 5 *μ*M Rhod2-AM or 4 *μ*M Fluo4-AM were determined by flow cytometry analysis of aliquots of 3 × 10^5^ cells. For fluorescence microscopy, the cells were grown on coating chamber slides and stained with 4 *μ*M Rhod2-AM or 4 *μ*M Fluo4-AM in media supplemented with 10% FBS. The cells were incubated for 30 min at 37°C. The cells were then washed in PBS, and images were acquired with confocal microscopy (Zeiss, Germany).

### 2.3. Flow Cytometry

For the cytometric analysis of cultured cell phenotypes, the cells were stained for 15 min at room temperature with fluorescein isothiocyanate-conjugated antibodies against human CD14, CD45, and HLA-DR (BD Biosciences); phycoerythrin-conjugated antibodies against human CD73 and CD166 (BD Biosciences); and CD105 (Serotec, Kidlington, UK). Corresponding isotype-matched mouse antibodies were used as controls. The cells were washed with PBS and fixed with 1% (*v*/*v*) paraformaldehyde (Sigma-Aldrich). The immunotype of the MSCs was determined by flow cytometry on a FACSCalibur instrument, and then the percentage of expressed cell surface antigens was calculated for 10,000 gated cell events.

### 2.4. Cell Differentiation

The cells were incubated under specific conditions to induce differentiation into osteocytes, chondrocytes, and adipocytes, and the multilineage potential was evaluated as previously described [34]. In brief, osteoblast or osteocyte formation was assessed by measuring the level of alkaline phosphatase (ALP; Sigma-Aldrich) activity. To confirm chondrogenic differentiation, cryosections were analyzed by safranin O staining (Sigma-Aldrich). In particular, to induce adipogenic differentiation, the cells were treated with an adipogenic medium consisting of high-glucose Dulbecco's modified Eagle's medium (Gibco) supplemented with 10% FBS, 0.5 mM 3-isobutyl-1-methylxanthine (Sigma), 1 mM dexamethasone (Sigma), 0.2 mm indomethacin (Sigma), and 10 mM h-insulin (Sigma) for 2 weeks. Assessment of adipocyte formation was based on the staining of accumulated lipid vacuoles with Oil red O (Sigma-Aldrich). Moreover, we used the fluorescent neutral lipid dye 4.4-difluoro-1, 3, 5, 7, 8-pentametyl-4-bora-3a, 4a-diaza-s-indacene (BODIPY 493/503, Molecular Probes, Carlsbad, California, USA) to confirm the lipid drop formation by confocal microscopy (Zeiss, Germany) [35]. Lipid vacuole accumulation was quantified by calculating the percentage of stained cells in the total population. All quantitation of stained cells was performed using SABIA software (Meetoo, Seongnam, Korea).

### 2.5. Western Blotting

Cell extracts were prepared in buffer containing 9.8 M urea, 4% CHAPS, 130 mM dithiothreitol, 40 mM Tris-HCl, and 0.1% sodium dodecyl sulfate (SDS). Protein concentrations were measured using a bicinchoninic acid kit (Sigma-Aldrich). Protein extracts (15 *μ*g) were separated by SDS-polyacrylamide gel electrophoresis, and the resolved proteins were transferred to nitrocellulose membranes. Each membrane was incubated with antibodies against Wnt5a, *β*-catenin (Abcam), phospho-GSK3*β* (Cell Signaling Technology, Danvers, MA, USA), PPAR*γ* (Santa Cruz Biotechnology), leptin (Thermo Fisher Scientific), and *β*-actin (Novus). The signals for the indicated proteins were detected with ChemiDoc™ MP Imaging System (Bio-Rad).

### 2.6. Quantitative Real-Time Polymerase Chain Reaction (qPCR) and Small Interfering RNA (siRNA)

qPCR was performed using a LightCycler™ 480 system (Roche, Mannheim, Germany). TaqMan probes were designed with the Universal ProbeLibrary Assay Design Center (Roche; see Supplementary Table 2) and used to quantitatively detect mRNA levels of the following genes: Wnt1, Wnt3, Wnt4, Wnt5a, Wnt5b, Wnt10b, PPAR*γ*, and leptin. Relative expression levels of the mRNAs of interest were calculated using the comparative threshold cycle method (2^−ΔΔCt^) with normalization to the *β*-actin mRNA expression level. Dharmacon (Chicago, IL, USA) designed the *β*-catenin siRNA and scrambled siRNA for use in the siRNA experiments. siRNAs were transfected using DharmaFECT reagent (Dharmacon) according to the manufacturer's instructions. The siRNA pools consisted of four different siRNA duplexes (see Supplementary Table 2).

### 2.7. Statistical Analyses

All data are reported as mean ± standard deviation and were analyzed in SPSS software (version 18). Significant differences were verified by one-way analysis of variance followed by the least significant difference post hoc test. The Student's *t*-test was used to compare data between two groups. *p* values less than 0.05 were considered statistically significant.

## 3. Results

### 3.1. Interindividual Differences in the Adipogenic Differentiation Potential of hUCB-MSCs

**Figure 1:**
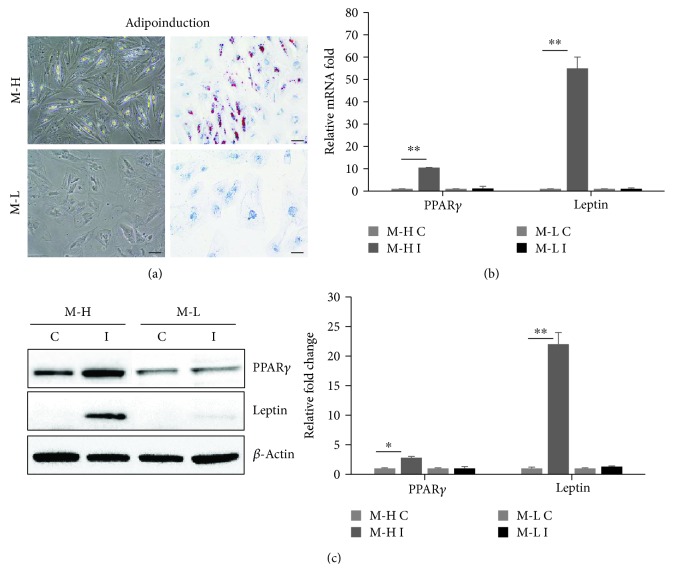
Two hUCB-MSC lines obtained from individual neonatal samples were cultured in adipogenic-specific medium. MSCs-H (M-H) and MSCs-L (M-L) were distinguished by high and low potential of adipogenic differentiation, respectively. (a) Adipogenic differentiation was examined by lipid vacuole formation and Oil red O staining (red), as indicated, suggesting no adipogenesis in MSCs-L. Oil red O staining was negative in MSCs-L versus MSCs-H at day 14 of induction. Scale bar = 50 *μ*m. (b) mRNA expression of the adipogenic markers PPAR*γ* and leptin was quantified by qPCR (mean ± SD, *n* = 3, ^∗∗^ *p* < 0.01). The expression levels of these genes were normalized to those of *β*-actin in control cells, which was defined as 1 (mean ± SD, *n* = 3, ^∗∗^ *p* < 0.01). (c) Protein expression of the adipogenic-specific markers PPAR*γ* and leptin was determined using Western blotting, with *β*-actin serving as a loading control. Expression levels were normalized to *β*-actin, with the expression levels in the control defined as 1 (right panel, mean ± SD, *n* = 3, ^∗∗^ *p* < 0.01, ^∗^ *p* < 0.05). C: control; I: induction.

Based on a previous report demonstrating variability in the adipogenic differentiation potential of hUCB-MSCs obtained from different donors [36], we further evaluated interindividual differences in the adipogenic differentiation potential of hUCB-MSCs obtained from six donors based on Oil red O activity (Supplementary Figure 1). To further investigate the mechanisms contributing to the differentiation potential of the hUCB-MSCs lots, we selected two representative hUCB-MSCs lots with high and low potential designated MSCs-H and MSCs-L, respectively. MSC-specific antigens largely did not differ among donors (Supplementary Figure 2A). The hUCB-MSCs lots also exhibited a similar effect with respect to multilineage differentiation ability, such as differentiation to osteoblasts (confirmed by ALP activity) and chondrogenesis (safranin O staining, Supplementary Figure 2B). However, the hUCB-MSCs lots demonstrated different degrees of adipogenic differentiation potential. After 14 days of adipogenic induction, we evaluated the intensity of Oil red O staining and lipid vacuole formation for further validation of the adipogenic differentiation of hUCB-MSCs, as well as the expression of the adipogenic-specific markers PPAR*γ* and leptin. As shown in Figure 1, MSCs-H showed extensive formation of lipid vacuoles, positive Oil red O staining, and strong expression of PPAR*γ* or leptin by qPCR or immunoblotting. By contrast, MSCs-L showed no lipid vacuole formation, negative Oil red O staining, and weak expression of PPAR*γ* or leptin. Collectively, our data confirmed that the adipogenic differentiation potential of hUCB-MSCs can vary markedly among different donors.

### 3.2. Intracellular Calcium Level Determines the Adipogenic Differentiation Potential of MSCs

**Figure 2:**
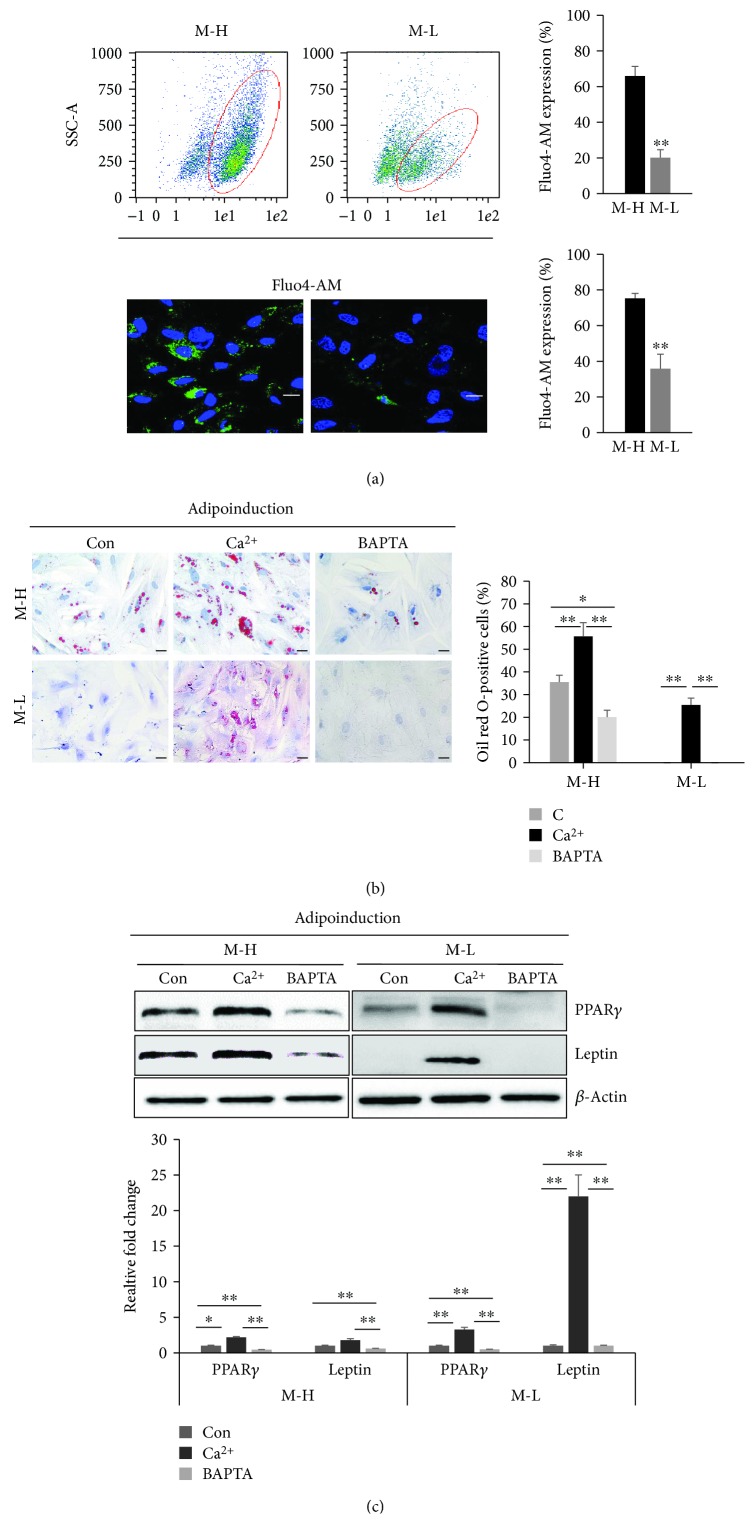
Effect of the intracellular calcium level on adipogenic differentiation of hUCB-MSCs with high or low adipogenic differentiation potential. (a) Fluo4-AM was used to measure the intracellular calcium level in the control condition from MSCs-H (M-H) or MSCs-L (M-L). Upper: the Fluo4-AM expression levels of calcium were measured by flow cytometry. The populations shown indicate the Fluo4-AM staining profile (red circle) versus the isotype control staining profile, and the percentage of Fluo4-AM-positive cells is shown (mean ± SD, *n* = 3, ^∗∗^ *p* < 0.01). Lower: the calcium levels were analyzed by fluorescence microscopy after Fluo4-AM staining (green). Nuclei were stained with 6-diamidino-2-phenylindole (DAPI; blue). The merged image is an overlay of the DAPI and Fluo4-AM images (scale bar = 50 *μ*m, mean ± SD, *n* = 3, ^∗∗^ *p* < 0.01). Confocal microscopy was used to determine the fluorescence intensity of the cytoplasmic Ca^2+^ level. (b, c) To assess the effect of Ca^2+^ or BAPTA-AM treatment on adipogenic differentiation, the cells cultured under each experimental condition were assessed for a differentiation period of 14 days. (b) Staining with Oil red O was significantly increased with Ca^2+^ treatment in MSCs-H and MSCs-L and was significantly decreased with BAPTA-AM treatment (mean ± SD, *n* = 3, ^∗∗^ *p* < 0.01, ^∗^ *p* < 0.05). Scale bar = 50 *μ*m. (c) Adipogenic-related proteins (PPAR*γ* and leptin) measured by immunoblotting, with *β*-actin serving as a loading control. Expression levels were normalized to *β*-actin, with the expression levels in the control defined as 1 (lower panel, mean ± SD, *n* = 3, ^∗∗^ *p* < 0.01, ^∗^ *p* < 0.05). BAPTA: BAPTA-AM.

Given that Ca^2+^ is a ubiquitous intracellular signal responsible for regulating numerous cellular processes important to differentiation [20], we evaluated whether the intracellular Ca^2+^ level contributes to the difference in the adipogenic differentiation potential between MSCs-H and MSCs-L based on the degree of staining of the Ca^2+^-sensitive dye Fluo4-AM and Rhod2-AM. By Fluo4-AM labeling, flow cytometry showed that under the control condition, the cytoplasmic level of Ca^2+^ was higher in MSCs-H (75.3 ± 2.8%) than in MSCs-L (35.9 ± 8.1%, Figure 2(a)). In addition, confocal microscopy showed that the fluorescence intensity of the cytoplasmic Ca^2+^ level was much higher in the MSCs-H (66.1 ± 5.3%) than in the MSCs-L (20.1 ± 4.5%, Figure 2(a)). Similarly, the Ca^2+^ level of MSCs-H was significantly higher than that of MSCs-L based on Rhod2-AM labeling (Supplementary Figure 3). Taken together, these results suggested that the intracellular Ca^2+^ level significantly differed between MSCs-H and MSCs-L. Moreover, a similar finding was obtained for BM-MSCs and AT-MSCs, which both showed greater adipogenic differentiation potential by Oil red O staining and also higher Ca^2+^ levels (>50%) than the hUCB-MSCs, as determined by flow cytometry (Rhod2-AM, Supplementary Figure 4).

Treatment of extracellular calcium has also been shown to increase the extent of calcium influx through the calcium channel [37]. Therefore, to examine the causative role of Ca^2+^ in hUCB-MSCs adipogenic differentiation, the cells were treated with calcium chloride (1.8 mM) to elevate the intracellular Ca^2+^ level during 5 days of culture (i.e., one passage culture of MSCs-H or MSCs-L, Supplementary Figure 5) [38]. Furthermore, the cells were pretreated with 5 *μ*M BAPTA-AM, which is a cell-permeant intracellular Ca^2+^ chelator, for 2 h. To analyze whether Ca^2+^ or BAPTA-AM treatment altered the MSC characteristics of the cells, the expressions of MSC-specific antigens and multilineage differentiation factors were compared in the different treatment groups (see Supplementary Figure 6). There was no difference in the expression of MSC-specific surface antigens among the control, Ca^2+^ treatment, or BAPTA-AM treatment groups (Supplementary Figure 6A). There was also no difference in the multilineage differentiation ability of the different groups, including differentiation to osteoblasts and chondrocytes as confirmed by ALP staining and safranin O staining, respectively (Supplementary Figure 6B). In particular, under identical adipogenic conditions, compared with the untreated control, Ca^2+^-treated cells showed significantly increased Oil red O staining in both MSCs-H and MSCs-L. In detail, larger lipid drops were formed in the Ca^2+^-treated MSCs-H compared to the control MSCs-H (Figure 2(b)). Moreover, for the MSCs-L, no lipid vacuole formation was observed in the untreated condition, whereas Ca^2+^-treated cells showed significantly elevated Oil red O activity at up to 28.7%. Specifically, in BODIPY 493/503 staining for adipogenic potential, Ca^2+^-treated cells showed significantly high levels of lipid vacuole formation compared with those observed in control MSCs-H and MSCs-L (Supplementary Figure 7). Similarly, the Ca^2+^-treated cells showed significantly enhanced expression of PPAR*γ* and leptin relative to that observed in the untreated control cells for both MSCs-H and MSCs-L based on immunoblotting (Figure 2(c)). However, pretreatment with BAPTA-AM inhibited the adipogenic potential compared to that of the control in both MSCs-H and MSCs-L (Figures 2(b) and 2(c)). Overall, the culture medium showed no accumulation of lipid vacuoles within cells from the untreated, Ca^2+^-treated, and BAPTA-AM-treated groups, which were analyzed by staining with Oil red O (Supplementary Figure 8). These data demonstrated that the adipogenic differentiation potential of hUCB-MSCs is largely controlled by Ca^2+^.

### 3.3. Wnt/*β*-Catenin Signaling Is Repressed during the Adipogenic Differentiation of hUCB-MSCs

**Figure 3:**
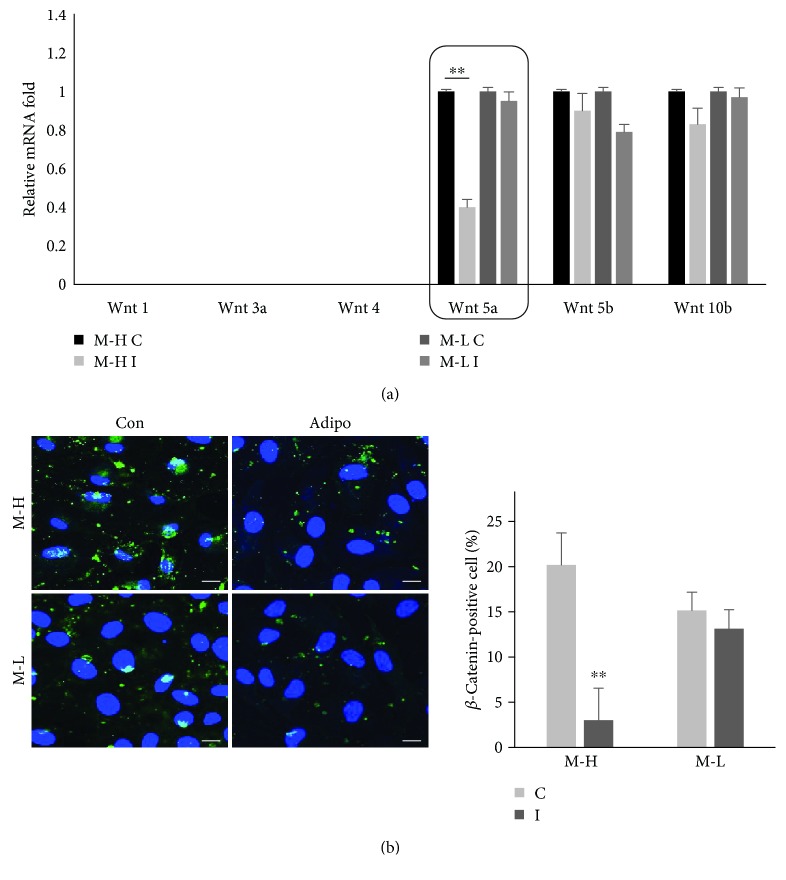
Validation of Wnt/*β*-catenin expression in MSCs-H or MSCs-L. (a) Cells cultured in adipogenic medium for 14 days. Expression of Wnt family members (Wnt1, Wnt3a, Wnt4, Wnt5a, Wnt5b, Wnt10b) in MSCs-H or MSCs-L was determined with qPCR (mean ± SD, *n* = 3, ^∗∗^ *p* < 0.01). Wnt5a expression showed a significant decline during the adipogenic induction of M-H (black box). The expression levels of these genes were normalized to those of *β*-actin in control cells, which was defined as 1. (b) Intracellular distribution and expression of *β*-catenin (green) assessed by quantifying the percentage of positively stained cells. Nuclei were stained with DAPI (blue). The merged image is an overlay of the DAPI and catenin images (scale bar = 50 *μ*m, mean ± SD, *n* = 3, ^∗∗^ *p* < 0.01). C: control; I: induction.

Wnt signaling maintains the cell growth of embryonic stem cells by stimulating cell division and inhibiting differentiation [39], and recent reports suggest that Wnt signaling also plays a main role in controlling the differentiation of MSCs from adult sources [40]. First, to determine whether Wnt signaling is related to the adipogenic differentiation in hUCB-MSCs, we examined the expression levels of Wnt family members (Wnt1, Wnt3a, Wnt4, Wnt5a, Wnt5b, and Wnt10b) with qPCR in the two hUCB-MSCs lots (MSCs-H and MSCs-L) after adipogenic differentiation. Only the mRNA level of Wnt5a was significantly decreased in MSCs-H by adipogenic induction (Figure 3(a)). Given that the Wnt/*β*-catenin pathway has been shown to maintain stem cells in a stemness and undifferentiated state, we next analyzed the degree of degradation of *β*-catenin, a key mediator of Wnt5a signaling, in MSCs-H in comparison with MSCs-L, based on the fluorescence intensity. Under control conditions of MSCs-H, *β*-catenin was distributed within the cytoplasm and was intensively localized in the plasma membrane, whereas the adipogenic cells had a decreased *β*-catenin fluorescence region within the cytoplasm (Figure 3(b)), suggesting an active role of the canonical Wnt pathway during adipogenic differentiation. To further confirm this result, we determined Wnt5a/*β*-catenin expression in MSCs-H or MSCs-L. As expected, the Western blot result showed that the Wnt5/*β*-catenin protein level of the MSCs-L was greater than that of the MSCs-H (Supplementary Figure 9). Taken together, these results indicated that activity of the canonical Wnt5a/*β*-catenin pathway is inhibited during the adipogenic differentiation of hUCB-MSCs, suggesting a role of this pathway in adipogenesis.

### 3.4. Ca^2+^ Augments the Adipogenic Effect through Suppression of Wnt/*β*-Catenin Signaling in hUCB-MSCs

**Figure 4:**
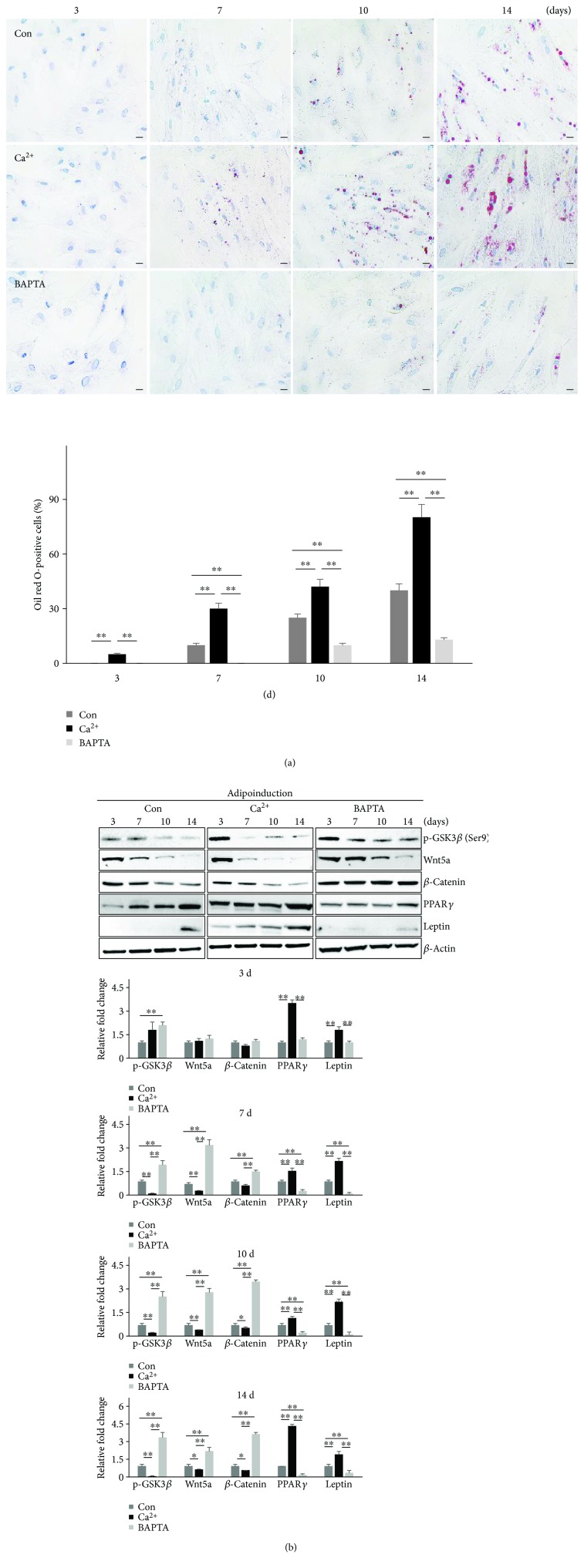
Effect of Ca^2+^ on adipogenic differentiation of MSCs-H. Cells were treated with Ca^2+^ or BAPTA-AM and then cultured in adipogenic-specific medium. Adipogenic differentiation potential was monitored at day 3, day 7, day 10, and day 14, respectively. (a) Cells were stained with Oil red O, and the percentage of Oil red O-positive cells is shown (scale bar = 50 *μ*m, mean ± SD, *n* = 3, ^∗∗^ *p* < 0.01). (b) Time course of protein expression of Wnt5a, *β*-catenin, and p-GSK3*β* in the Wnt5a/*β*-catenin signaling pathway and of PPAR*γ* and leptin in adipogenic signaling determined by Western blot, with *β*-actin serving as a loading control. Expression levels were normalized to *β*-actin, with the expression levels in the control defined as 1 (lower panel, mean ± SD, *n* = 3, ^∗∗^ *p* < 0.01, ^∗^ *p* < 0.05). BAPTA: BAPTA-AM.

**Figure 5:**
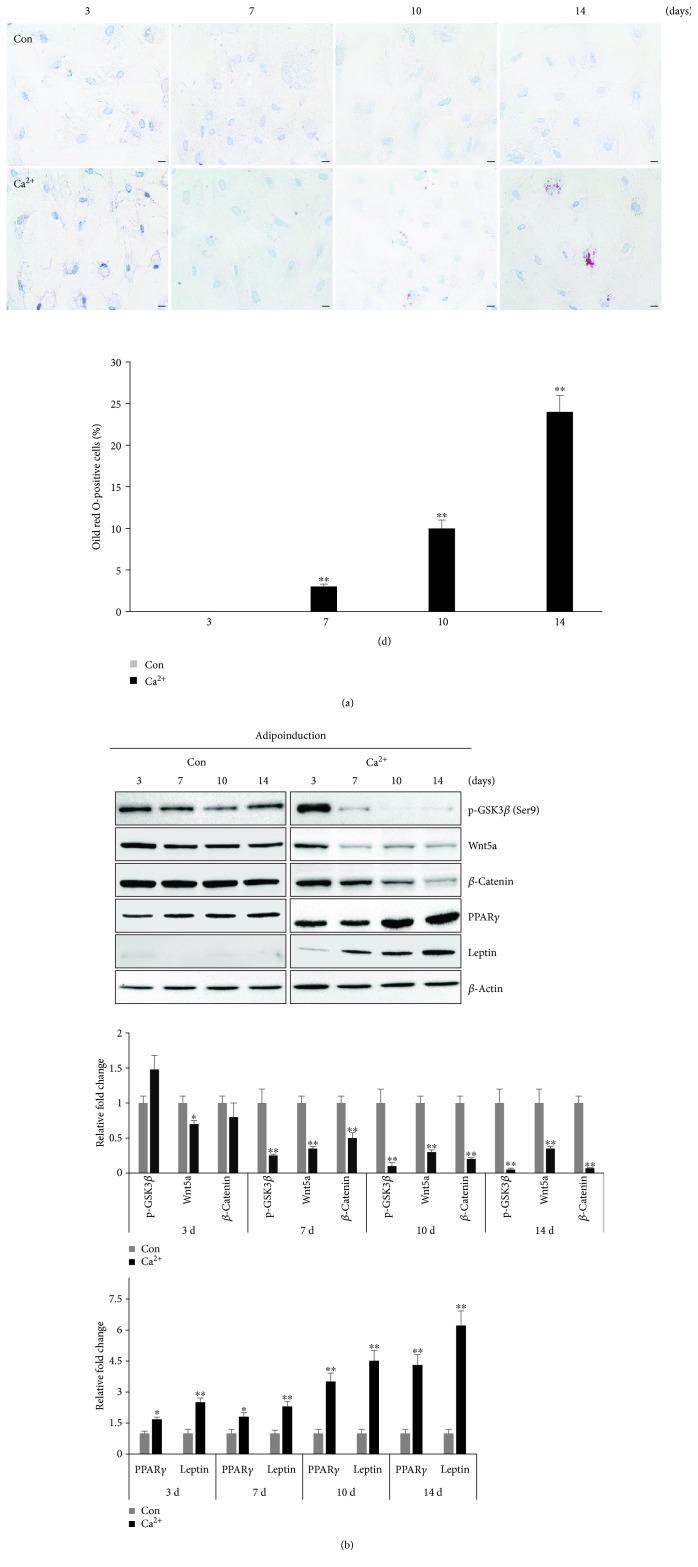
Effect of Ca^2+^ on the adipogenic differentiation of MSCs-L. Cells were treated with Ca^2+^ and then cultured in adipogenic-specific medium. Adipogenic differentiation potential was monitored at day 3, day 7, day 10, and day 14, respectively. (a) Cells were stained with Oil red O, and the percentage of Oil red O-positive cells is shown (scale bar = 50 *μ*m, mean ± SD, *n* = 3, ^∗∗^ *p* < 0.01). (b) Time course of protein expression of Wnt5a, *β*-catenin, and p-GSK3*β* in the Wnt5a/*β*-catenin signaling pathway and of PPAR*γ* and leptin in the adipogenic signaling pathway determined by Western blot analysis, with *β*-actin serving as a loading control. Expression levels were normalized to *β*-actin, with the expression levels in the control defined as 1 (lower panel, mean ± SD, *n* = 3, ^∗∗^ *p* < 0.01, ^∗^ *p* < 0.05).

Based on the results described above, we hypothesized that adipogenic potential may be enhanced through Ca^2+^ to actively control the Wnt5a/*β*-catenin pathway. To test this hypothesis, we continuously monitored the differentiation ability and activation of the Wnt5a/*β*-catenin pathway in MSCs-H or MSCs-L over time at days 3, 7, 10, and 14 of adipogenic differentiation. Under the control condition of MSCs-H, the accumulation of lipid droplets (based on Oil red O or BODIPY 493/503 staining) significantly increased from day 3 to day 14. The adipogenic potential of Ca^2+^-treated cells was significantly increased at day 7 and remained elevated at days 10 and 14 compared to that observed under the control condition. In addition, pretreatment with BAPTA-AM significantly inhibited the adipogenic differentiation potential compared to that of the control (Figure 4(a), Supplementary Figure 10). Phosphorylation of *β*-catenin by GSK3*β* results in its degradation, which promotes the inactivation of Wnt/*β*-catenin signaling [41]. Since phosphorylation of GSK3*β* at Ser9 can lead to GSK3*β* inactivation [42], we also examined the effect of Ca^2+^ on the phosphorylation of GSK3*β* with Western blot analysis. Under the three conditions, the expression of phosphorylated GSK3*β* (p-GSK3*β*, Ser9), Wnt5a, and *β*-catenin was gradually downregulated during adipogenic differentiation, while the expression of adipogenesis-related proteins (PPAR*γ* and leptin) was upregulated. The expression levels of p-GSK3*β*, Wnt5a, and *β*-catenin were also lower in the Ca^2+^-treated cells to those of the control and BAPTA-AM-treated groups. In addition, the Ca^2+^-treated cells strongly expressed PPAR*γ* and leptin, but these proteins were only weakly expressed in the BAPTA-AM-treated group. Similar to the Oil red O staining pattern, leptin expression, as a late adipogenesis marker, was upregulated after 7 days of induction in the Ca^2+^ treatment group but not in the control and BAPTA-AM treatment groups. PPAR*γ* expression, as a transcription marker of adipogenesis, was already induced after only 3 days in the Ca^2+^ treatment group. Wnt5a and *β*-catenin expression levels were lower in the Ca^2+^-treated cells than in the control and BAPTA-AM groups, with a significant difference noted on day 7 of induction (Figure 4(b)). For the MSCs-L, under adipogenic conditions, significantly larger lipid drops were formed within the Ca^2+^-treated cells than control cells. Specifically, Oil red O staining was significantly activated from day 7 to day 14 in the Ca^2+^-treated cells, whereas almost no cells in the control group were stained with Oil red O until 14 days. Indeed, the differentiation rate of Ca^2+^-treated cells was much greater than that of the control (Figure 5(a)). Similarly, the immunoblotting analysis showed no change of PPAR*γ* expression and no leptin expression in the control. In addition, the Ca^2+^-treated cells showed significantly decreased expression levels of p-GSK3*β*, Wnt5a, and *β*-catenin relative to those observed in control cells at all time points (Figure 5(b)). These data suggest that treatment with Ca^2+^ induced the adipogenic differentiation of hUCB-MSCs through a Wnt5a/*β*-catenin-dependent signaling pathway.

### 3.5. A Ca^2+^-Based System Has a Synergic Effect on Adipogenic Potential via Negatively Regulating the Wnt5a/*β*-Catenin Pathway in hUCB-MSCs

We tested the correlation between Wnt5a expression and the adipogenic potential of hUCB-MSCs by activating or inhibiting Wnt5a, as confirmed by evaluation of the protein levels of Wnt5a or Dkk-1. Dkk-1 is a secreted protein that functions as a negative regulator of Wnt signaling [43]. MSCs-H treated with Dkk-1 showed significantly increased numbers of lipid vacuoles at day 14 of adipogenic differentiation compared to those in the control condition. In addition, treatment with Wnt5a significantly blocked the adipogenic differentiation potential compared to that of the control. In MSCs-L, lipid vacuoles were only formed with Dkk-1 treatment, further suggesting that Wnt5a plays a key role in the adipogenic differentiation of hUCB-MSCs (Supplementary Figure 11A).

To confirm that *β*-catenin functionally contributed to the adipogenic potential of hUCB-MSCs, we blocked *β*-catenin expression using specific siRNA. Control experiments showed that treatment with the target siRNA effectively inhibited *β*-catenin expression at the protein level, as confirmed by immunoblotting, and this suppression was maintained for up to 15 days (Supplementary Figure 11B). In both MSCs-H and MSCs-L, silencing of *β*-catenin resulted in increased Oil red O staining during adipogenic induction compared with that detected in naïve cells or scrambled siRNA-transfected cells (Supplementary Figure 11C). Taken together, these data demonstrate that inhibiting *β*-catenin accelerated the adipogenic potential of hUCB-MSCs.

**Figure 6:**
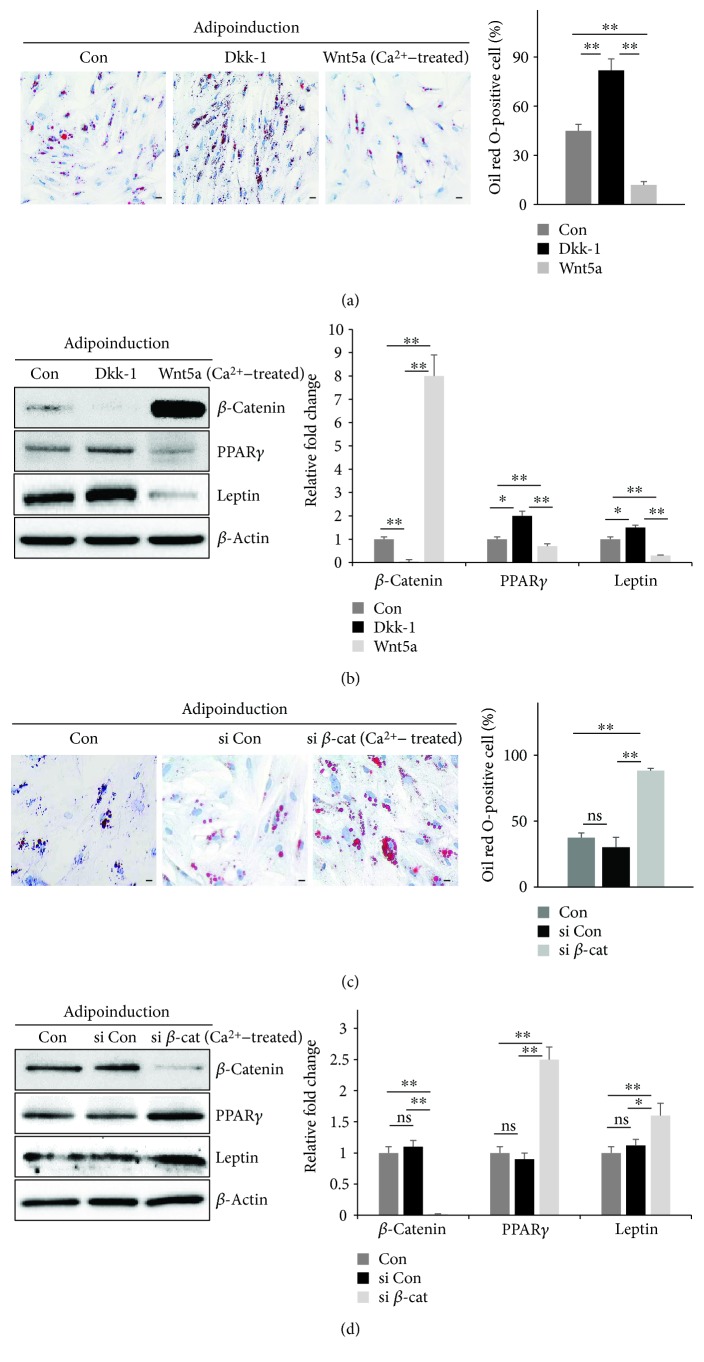
Ca^2+^-based system has a synergic effect on adipogenic potential via negatively regulating the Wnt5a/*β*-catenin pathway in hUCB-MSCs. (a, b) MSCs-L were treated with Ca^2+^, and then a Wnt activator (100 ng/mL Wnt5a) or inhibitor (50 ng/mL Dkk-1) was added to the adipogenic medium for the initial 4 days of induction. (a) At day 14 of adipogenic induction, the cells were stained with Oil red O, and activity was quantified by counting the positively stained cells (scale bar = 50 *μ*m, mean ± SD, *n* = 3, ^∗∗^ *p* < 0.01). (b) Immunoblotting analysis was used to detect Wnt5a/*β*-catenin signaling and adipogenic markers in Ca^2+^-treated MSCs-L with Wnt5a or Dkk-1, with *β*-actin serving as a loading control. (c, d) Inhibition of *β*-catenin induced adipogenic differentiation in MSCs-L treated with Ca^2+^. MSCs-L were transfected with scramble siRNA (si Con) or *β*-catenin siRNA (si *β*-cat). (c) Cells were stained with Oil red O, and activity was quantified by counting the positively stained cells (scale bar = 50 *μ*m, mean ± SD, *n* = 3, ^∗∗^ *p* < 0.01). (d) The expression levels of Wnt5a/*β*-catenin signaling and adipogenic-related markers were measured using immunoblotting analysis. *β*-Actin was used at the loading control. (b, d) Expression levels were normalized to *β*-actin, with the expression levels in the control defined as 1 (right panel, mean ± SD, *n* = 3, ^∗∗^ *p* < 0.01, ^∗^ *p* < 0.05). ns: not significant.

To further examine the mechanistic relationship between Wnt5a/*β*-catenin and adipogenic differentiation induced by Ca^2+^, the synergistic potential of Ca^2+^, Wnt5a, Dkk-1, and *β*-catenin was evaluated in MSCs-L. Oil red O activities were significantly increased in the Dkk-1 treatment group in the presence of Wnt5a or Dkk-1 after differentiation. In contrast, in Wnt5a-treated cells, the number of lipid droplets decreased to 30% that of the control (Figure 6(a)). This finding was corroborated by Western blot analysis as the Dkk-1-treated cells also showed reduced protein expression of Wnt5a and *β*-catenin, indicating that adipogenesis markers are reduced by Wnt5a treatment and elevated by Dkk-1 treatment (Figure 6(b)). *β*-Catenin siRNA treatment significantly increased the Oil red O activity to 50% greater than the level detected in the control group (Figure 6(c)) and significantly increased the protein levels of PPAR*γ* and leptin (Figure 6(d)). In addition, *β*-catenin expression was markedly downregulated under *β*-catenin suppression with siRNA. Thus, we conclude that Wnt5a/*β*-catenin is a main signal for adipogenic differentiation in hUCB-MSCs. Moreover, these findings suggest that a Ca^2+^-based system has a synergic effect on the adipogenic potential via negatively regulating the Wnt5a/*β*-catenin pathway in hUCB-MSCs.

## 4. Discussion

In the present study, hUCB-MSCs collected from different donors were classified according to the extent of adipogenic differentiation, which confirmed previous reports of the high donor-to-donor variability in the adipogenic differentiation potential of hUCB-MSCs [17, 35].

A recent study demonstrated the effect of calcium in the early stage of adipocyte differentiation and the underlying mechanism [21, 22]. During adipocyte differentiation based on changes in the intracellular calcium concentration, the PPAR*γ* transcription factor acts as the adipocyte master regulator that is activated in cascades to induce adipocyte differentiation and regulate the expression of various genes that accelerate adipose accumulation [21, 22]. Based on this finding, we explored whether the intracellular calcium level is a primary cause of this variation in adipogenic differentiation potential among donors. Indeed, we found a significant difference in the intracellular calcium level between two representative hUCB-MSCs lots with high and low adipogenic potential, MSCs-H and MSCs-L, that were induced for adipogenic differentiation. Furthermore, the adipogenic differentiation of both MSCs-H and MSCs-L improved with supplementation of calcium, whereas treatment with the calcium chelator BAPTA-AM led to a dramatic decrease in the adipogenic differentiation potential. Moreover, calcium increased the rate of adipogenic differentiation. Thus, the variation of intracellular calcium levels of hUCB-MSCs from different donors appears to influence the actual adipogenic differentiation regulation. Thus, intracellular calcium can be a marker for predicting the extent of the adipogenic differentiation of hUCB-MSCs.

Previous studies have shown that the adipogenic differentiation potential of MSCs from fetal-derived sources (amniotic fluid, placenta, UC, and UCB) was significantly lower than those derived from adult tissues (AT and BM) [44], which suggests an association between adipogenic differentiation potential and aging [45]. We further showed that the intracellular calcium levels of AT-MSCs and BM-MSCs (with high adipogenic differentiation potential) were higher than those of hUCB-MSCs. A recent report demonstrated an association between intracellular calcium concentration and senescence [46]. Thus, it may be conjectured that the intracellular calcium concentration is low in fetal-derived MSCs with low senescence, including hUCB-MSCs, which contributes to their relatively low adipogenic differentiation potential. Further investigation of the association between the adipogenic differentiation potential of hUCB-MSCs and the age or senescence degree of donors is expected to provide a clue that can explain the individual variation in the adipogenic differentiation potential of hUCB-MSCs.

Wnt, a secreted protein, is a cellular signal transduction pathway that determines the fate of the cell and regulates its polarity, proliferation, and differentiation [23, 24]. Specifically, canonical Wnt determines the cell fate and regulates proliferation, whereas noncanonical Wnt regulates cell polarity. The canonical Wnt pathway is regulated by calcium or small G-proteins whose target genes are induced by the level of *β*-catenin in the cytoplasm [47]. Recently, canonical Wnt and noncanonical Wnt have been distinguished based on *β*-catenin signal transduction [48]. PPAR*γ* is a main transcription factor that leads to adipocyte differentiation, and its increased expression induces the expression of adipocyte-specific genes such as aP2 and leptin, thereby forming an adipocyte with distinct morphology and function [50, 51]. Wnt10b/*β*-catenin, an intracellular regulator, inhibits the expression of PPAR*γ* to suppress the expression of adipocyte-specific genes, causing interrupted adipocyte formation [29, 51]. Similarly, the level of Wnt3a or Wnt10b, involved in the canonical pathway, is significantly reduced and *β*-catenin activation is decreased when AT-MSCs differentiate into adipocytes [52]. However, there is little information available on the association between Wnt and the adipogenic differentiation of fetal-derived MSCs, including hUCB-MSCs. In the present study, we found that only the level of Wnt5a decreased during the adipogenic differentiation of MSCs-H, whereas no changes in Wnt3 and Wnt10b were confirmed in hUCB-MSCs. This suggests that the adipogenic differentiation of hUCB-MSCs involves a different mechanism compared to that of other MSCs. Wnt5a is described as the representative member of the canonical Wnt pathway [53]. In the present study, we further observed reduced *β*-catenin levels only during the adipogenic differentiation of MSCs-H, associated with low expression of *β*-catenin in the cytoplasm. Similar to previous studies [41], we confirmed that Wnt5a/*β*-catenin is linked to a reduction in GSK3 phosphorylation. Moreover, Dkk-1 protein, which does not bind to Wnt but induces the inhibition of Wnt signaling [43], significantly enhanced adipogenic differentiation. In addition, adipogenic differentiation was improved after suppressing *β*-catenin with siRNA. Collectively, these results indicate that the adipogenic differentiation of hUCB-MSCs is induced by negative regulation of Wnt5a/*β*-catenin signaling.

**Figure 7:**
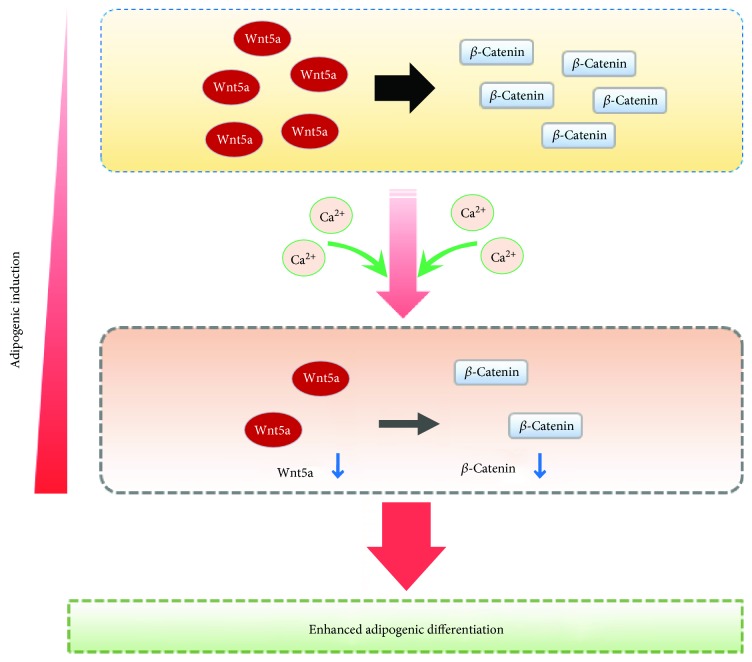
Schematic diagram of enhancing effect of calcium on adipogenic differentiation of hUCB-MSCs. The adipogenic differentiation of hUCB-MSCs is induced by decreased canonical Wnt5a/*β*-catenin signaling. Calcium accelerated the reduction of Wnt5a and *β*-catenin proteins during adipogenic induction, which resulted in significantly decreased Wnt5a/*β*-catenin signaling. Consequently, the exogenous calcium treatment enhanced the adipogenic differentiation via negatively regulating the Wnt5a/*β*-catenin signaling.

We further demonstrated that calcium accelerates the rate of Wnt5a/*β*-catenin signal reduction during the progression of adipogenic differentiation. Moreover, the adipogenic differentiation of calcium-treated MSCs-L increased with Dkk-1 as well as with inhibition of *β*-catenin in MSCs-L. However, these results are different with recent report showing a positive correlation between the calcium concentration and Wnt5a/*β*-catenin, and that the differentiation of skin cells is strongly induced by increased Wnt5a/*β*-catenin signal [54]. Therefore, our findings newly suggest that calcium can enhance adipogenic differentiation via negatively regulating Wnt5a/*β*-catenin signaling (Figure 7).

## 5. Conclusions

Overall, these results suggest that the intracellular calcium level can be a marker to predict the adipogenic differentiation potential of MSCs to improve the selection of MSCs for lipogenesis therapy. Importantly, these findings can be directly exploited to improve the application of hUCB-MSCs as a therapy for adipose tissue regeneration. Therefore, clinical application of the cells directly after exposure to calcium could improve the adipogenic potential and thus the therapeutic efficacy.

## Data Availability

The datasets generated during the current study are available from the corresponding author on reasonable request.

## Supplementary Materials

Supplementary Materials Supplementary Figure 1: donor-dependent adipogenic differentiation potential of hUCB-MSCs. Supplementary Figure 2: characterization of two hUCB-MSC lines. Supplementary Figure 3: the intracellular calcium level of MSCs-H and MSCs-L. Supplementary Figure 4: intracellular calcium level during the adipogenic differentiation of BM- and AT-MSCs. Supplementary Figure 5: intracellular calcium levels of MSCs-H and MSCs-L under treatment with Ca^2+^ or BAPTA-AM. Supplementary Figure 6: characterization of MSCs-H by Ca^2+^ or BAPTA-AM treatment. Supplementary Figure 7: effect of the intracellular calcium level on adipogenic differentiation from MSCs-H and MSCs-L. Supplementary Figure 8: analysis of adipogenic potentials. Supplementary Figure 9: the expression of Wnt5a/*β*-catenin from MSCs-H and MSCs-L. Supplementary Figure 10: effect of Ca^2+^ on adipogenic differentiation of MSCs-H by BODIPY 493/503 staining. Supplementary Figure 11: role of Wnt5a/*β*-catenin signaling in adipogenic differentiation. Supplementary Table 1: detailed information regarding the hUCB-MSCs used in this study. Supplementary Table 2: sequences of primers used for sequencing indicated the target genes.
