# Supplementary material for: Intracellular Calcium Determines the Adipogenic Differentiation Potential of Human Umbilical Cord Blood-Derived Mesenchymal Stem Cells via the Wnt5a/β-Catenin Signaling Pathway
Source: Stem Cells Int. 2018 Jul 11;2018:6545071. doi: 10.1155/2018/6545071 (PMC6079381; doi:10.1155/2018/6545071)
Supplement: Supplementary Materials — Supplementary Figure 1: donor-dependent adipogenic differentiation potential of hUCB-MSCs. Supplementary Figure 2: characterization of two hUCB-MSC lines. Supplementary Figure 3: the intracellular calcium level of MSCs-H and MSCs-L. Supplementary Figure 4: intracellular calcium level during the adipogenic differentiation of BM- and AT-MSCs. Supplementary Figure 5: intracellular calcium levels of MSCs-H and MSCs-L under treatment with Ca2+ or BAPTA-AM. Supplementary Figure 6: characterization of MSCs-H by Ca2+ or BAPTA-AM treatment. Supplementary Figure 7: effect of the intracellular calcium level on adipogenic differentiation from MSCs-H and MSCs-L. Supplementary Figure 8: analysis of adipogenic potentials. Supplementary Figure 9: the expression of Wnt5a/β-catenin from MSCs-H and MSCs-L. Supplementary Figure 10: effect of Ca2+ on adipogenic differentiation of MSCs-H by BODIPY 493/503 staining. Supplementary Figure 11: role of Wnt5a/β-catenin signaling in adipogenic differentiation. Supplementary Table 1: detailed information regarding the hUCB-MSCs used in this study. Supplementary Table 2: sequences of primers used for sequencing indicated the target genes. [file 6545071.f1.docx]

**Supplementary Materials**

**Intracellular calcium determines the adipogenic differentiation potential of human umbilical cord blood-derived mesenchymal stem cells via the Wnt5a/β-catenin signaling pathway**

Yun Kyung Bae^†^, Ji Hye Kwon^†^, Miyeon Kim, Gee-Hye Kim, Soo Jin Choi, Wonil Oh, Yoon Sun Yang, Hye Jin Jin* & Hong Bae Jeon*

Biomedical Research Institute, MEDIPOST Co., Ltd., Seongnam 13494, Korea

**Supplementary Figure 1. Donor-dependent adipogenic differentiation potential of hUCB-MSCs.**

Six hUCB-MSCs obtained from individual neonatal samples were cultured in adipogenic-specific medium for 14 days and stained with Oil red O. Scale bar = 50 μm.


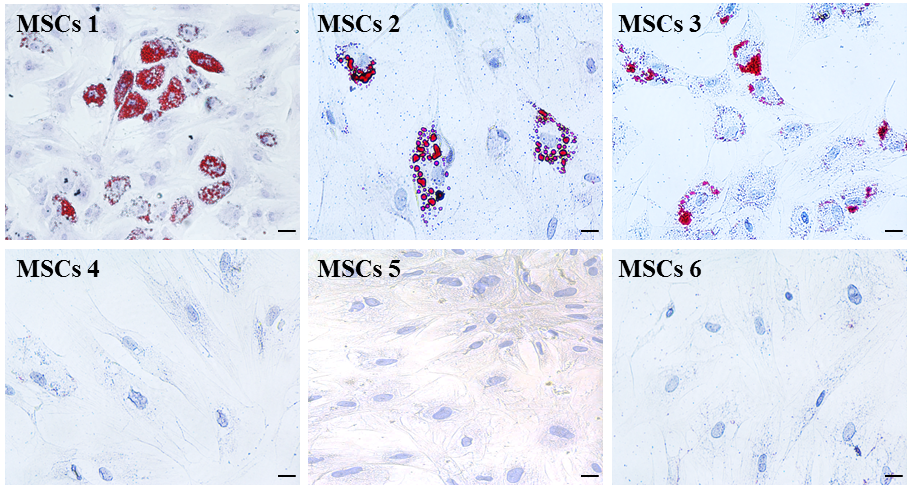


**Supplementary Figure 2. Characterization of two hUCB-MSC lines.**

(A) Both MSCs-H and MSCs-L were positive for expression of CD73, CD105, and CD166, but negative for CD14, CD45, and HLA-DR expression. Direct comparison of MSCs-H (M-H) and MSCs-L (M-L) demonstrated a similar pattern of surface markers. M2 indicates positive percentage of each marker (red line). (B) The level of differentiation to the osteogenic and chondrogenic lineages of the two MSC lines analyzed by staining with ALP and Safranin O, respectively. Scale bar = 50 μm.

**
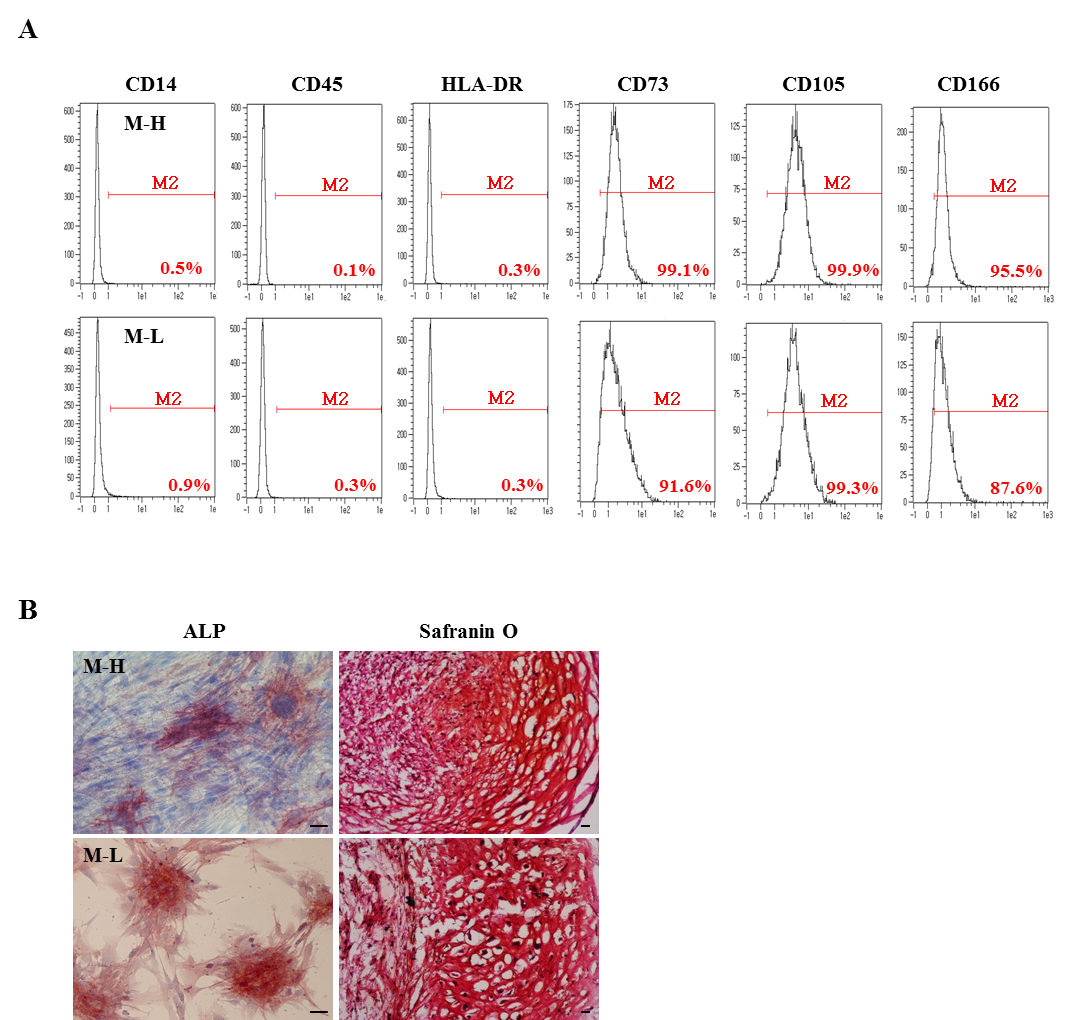
**

**Supplementary Figure 3. The intracellular calcium level of MSCs-H and MSCs-L.** Rhod2-AM (excitation wavelength, 552 nm; emission wavelength, 581 nm) was used to measure the intracellular calcium level in the control condition from MSCs-H (M-H) or MSCs-L (M-L). Upper: The Rohd2-AM expression levels of calcium were measured by flow cytometry. The populations shown indicate the Rhod2-AM staining profile (red box) vs. the isotype control staining profile, and the percentage of Rhod2-AM-positive cells is shown (mean ± SD, *n* = 3; ***p* < 0.01). Lower: The calcium levels were analyzed by fluorescence microscopy after Rhod2-AM staining (red). Nuclei were stained with DAPI (blue). The merged image is an overlay of the DAPI and Rhod2-AM images (Scale bar = 50 μm, mean ± SD, n = 3, ***p* < 0.01).


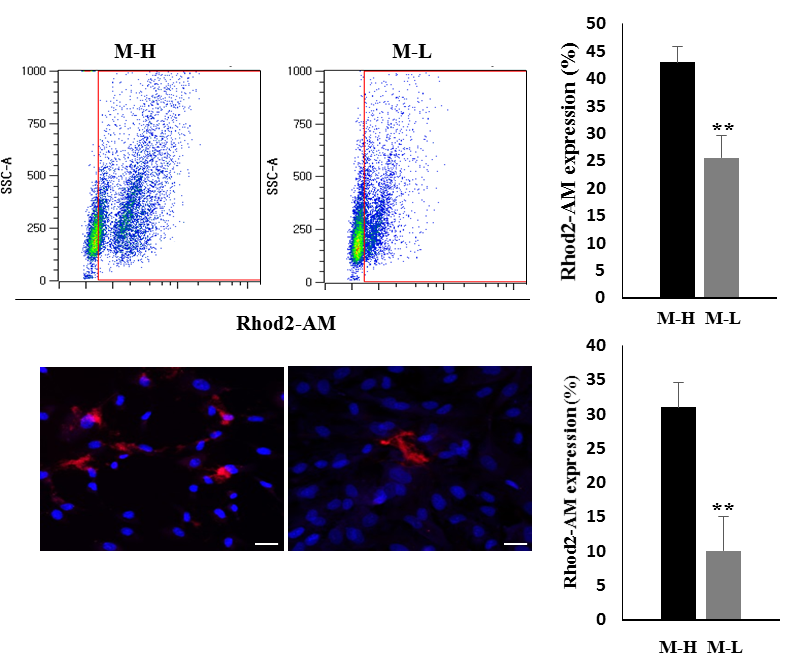


**Supplementary Figure 4. Intracellular calcium levels during the adipogenic differentiation of BM- and AT-MSCs.** (A) Both BM- and AT-MSCs were cultured in adipogenic-specific medium for 14 days and then stained with Oil red O. Scale bar = 50 μm. (B) The Rhod2-AM expression levels of calcium were measured by flow cytometry. The populations shown indicate the Rhod2-AM staining profile (red circle) vs. the isotype control staining profile, and the percentage of Rhod2-AM-positive cells is shown.


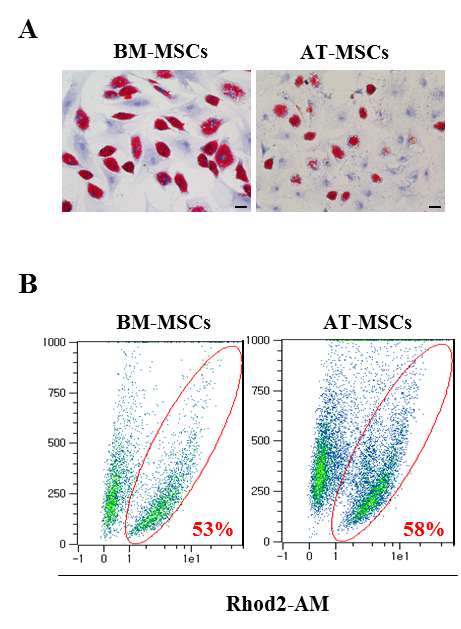


**Supplementary Figure 5.** **Intracellular calcium levels of MSCs-H and MSCs-L under treatment with Ca^2+^ or BAPTA-AM.** The Rhod2-AM expression levels of calcium were measured by flow cytometry. The populations shown indicate the Rhod2-AM staining profile (red circle), and the percentage of Rhod2-AM-positive cells is shown. Abbreviations: BAPTA, BAPTA-AM.


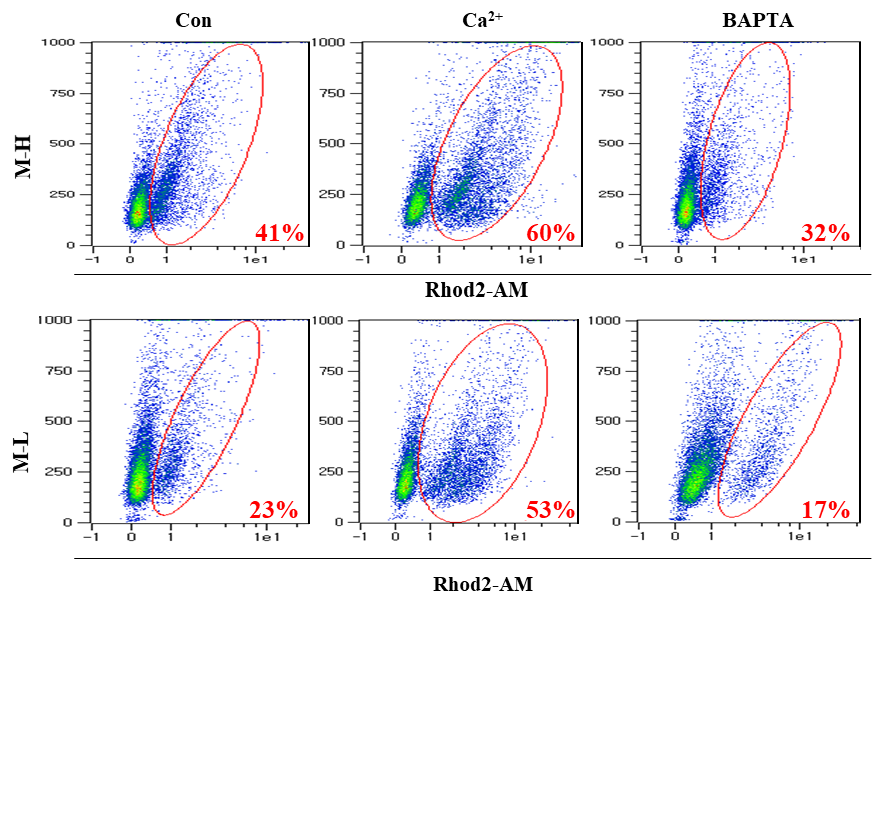


**Supplementary Figure 6. Characterization of MSCs-H by Ca^2+^ or BAPTA-AM treatment.**

(A) Cells in all three conditions showed positive expression of CD73, CD105, and CD166, but negative expression of CD14, CD45, and HLA-DR. M2 indicates the positive percentage for each marker (red line). (B) The level of osteogenic or chondrogenic differentiation potential was analyzed by staining with ALP and Safranin O, respectively. Scale bar = 50 μm. Abbreviations: BAPTA, BAPTA-AM.

**
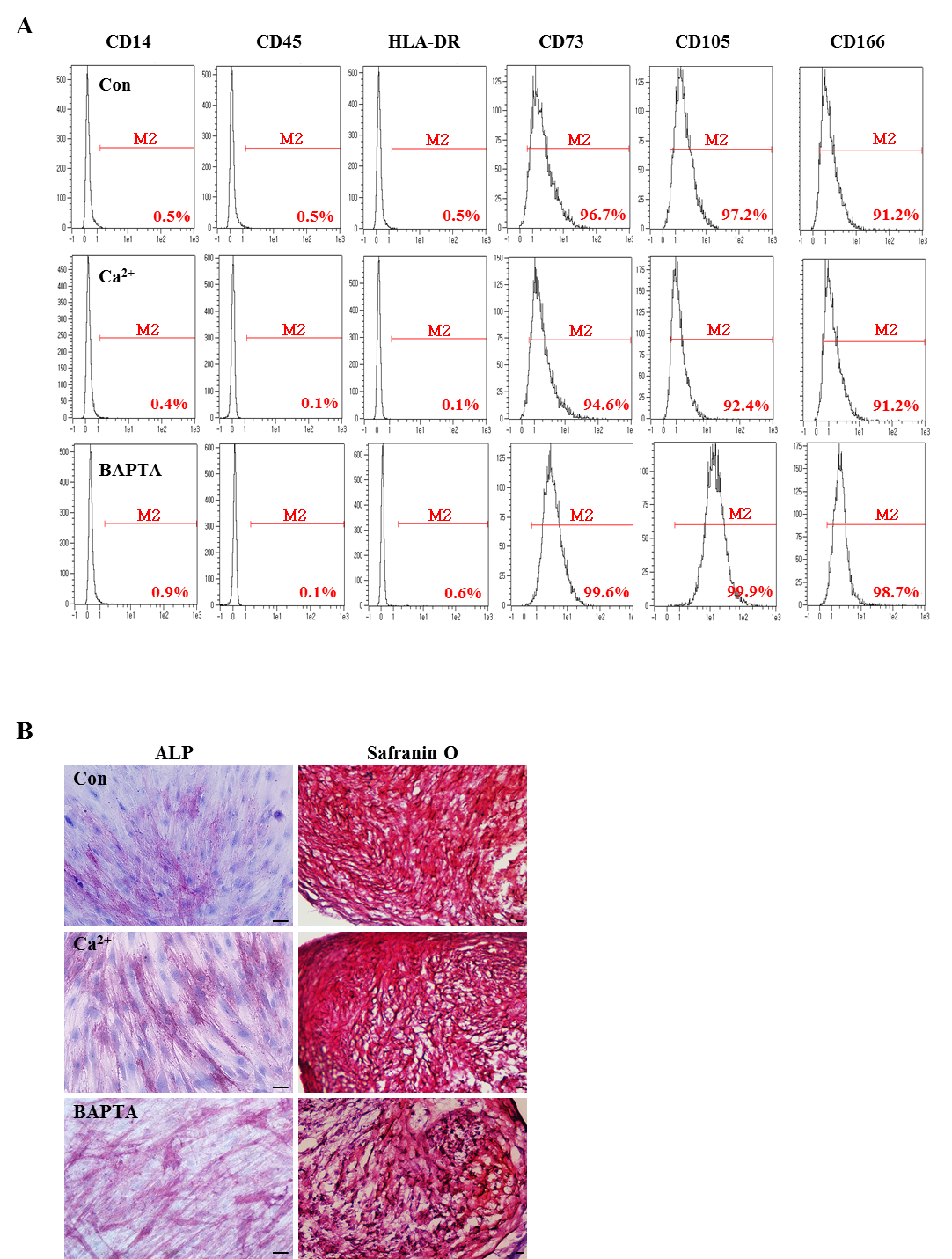
**

**Supplementary Figure 7.** **Effect of the intracellular calcium level on adipogenic differentiation from MSCs-H and MSCs-L.** To assess the effect of Ca^2+^ or BAPTA-AM treatment on adipogenic differentiation, the cells cultured under each experimental condition were assessed for a differentiation period of 14 days. The extent of lipid drop formation was analyzed by fluorescence microscopy after BIODIPY 493/503 staining (green). Nuclei were stained with DAPI (blue). The merged image is an overlay of the DAPI and BODIPY 493/503 images. Staining with BODIPY 493/503 was significantly increased with Ca^2+^ treatment in MSCs-H and MSCs-L, and was significantly decreased with BAPTA-AM treatment (mean ± SD, *n* = 3; ***p* < 0.01). Scale bar = 50 μm. Abbreviations: BAPTA, BAPTA-AM; BODIPY, BODIPY 493/503.


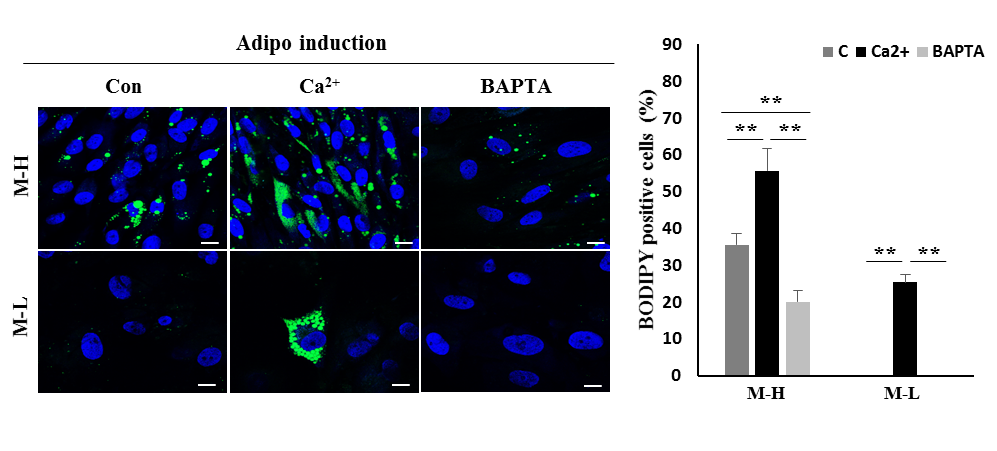


**Supplementary Figure 8.** **Analysis of adipogenic potential.** Both MSCs-H and MSCs-L of culture in conditioned medium with or without Ca^2+^, BAPTA-AM for 14 days. Adipogenic differentiation was examined by lipid vacuole formation observation and Oil red O staining, suggesting no adipogenic in all conditions. Scale bar = 50 μm. Abbreviations: BAPTA, BAPTA-AM.

**
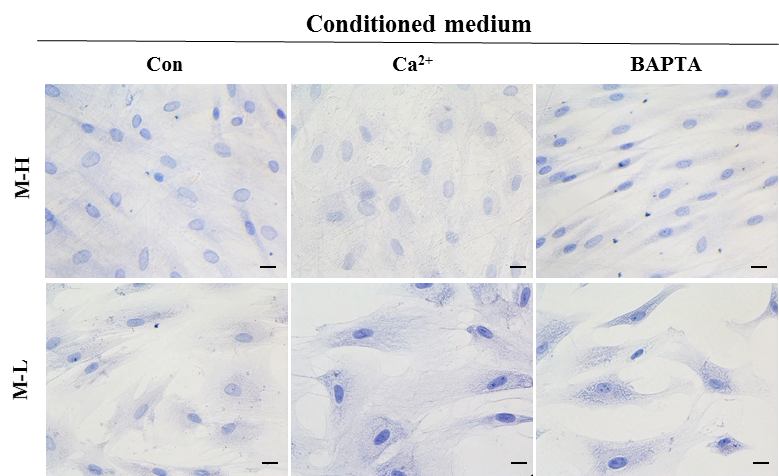
**

**Supplementary Figure 9.** **The expression of Wnt5a/β-catenin from MSCs-H and MSCs-L.**

Protein expression of Wnt5a/β-catenin determined using western blotting, with β-actin serving as a loading control. Expression levels were normalized to β-actin, with the expression levels in control defined as 1 (right panel; mean ± SD, *n* = 3; ***p* < 0.01).


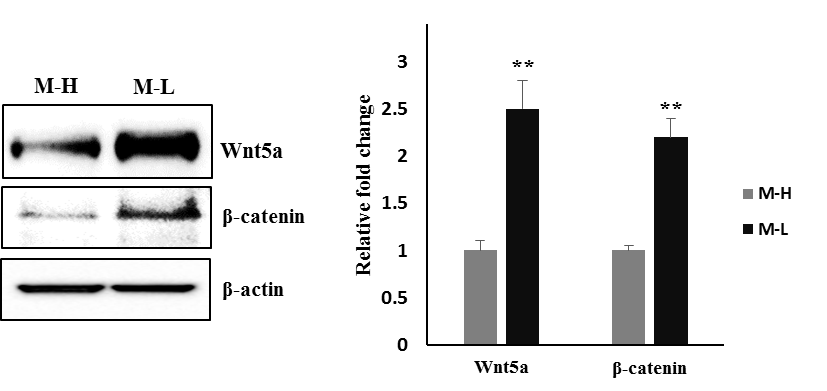


**Supplementary Figure 10.** **Effect of Ca^2+^ on the adipogenic differentiation of MSCs-H by BODIPY493/503 staining.** Cells were treated with Ca^2+^ or BAPTA-AM and then cultured in adipogenic-specific medium. Adipogenic differentiation potential was monitored at day 3, 7, 10, and 14, respectively. Lipid drop formation was analyzed by fluorescence microscopy after BODIPY 493/503 staining (green). Nuclei were stained with DAPI (blue). The merged image is an overlay of the DAPI and BIODIPY images, and the percentage of BODIPY-positive cells is shown (Scale bar = 50 μm, mean ± SD, n = 3, ***p* < 0.01, **p* < 0.05). Abbreviations: BODIPY, BODIPY 493/503.


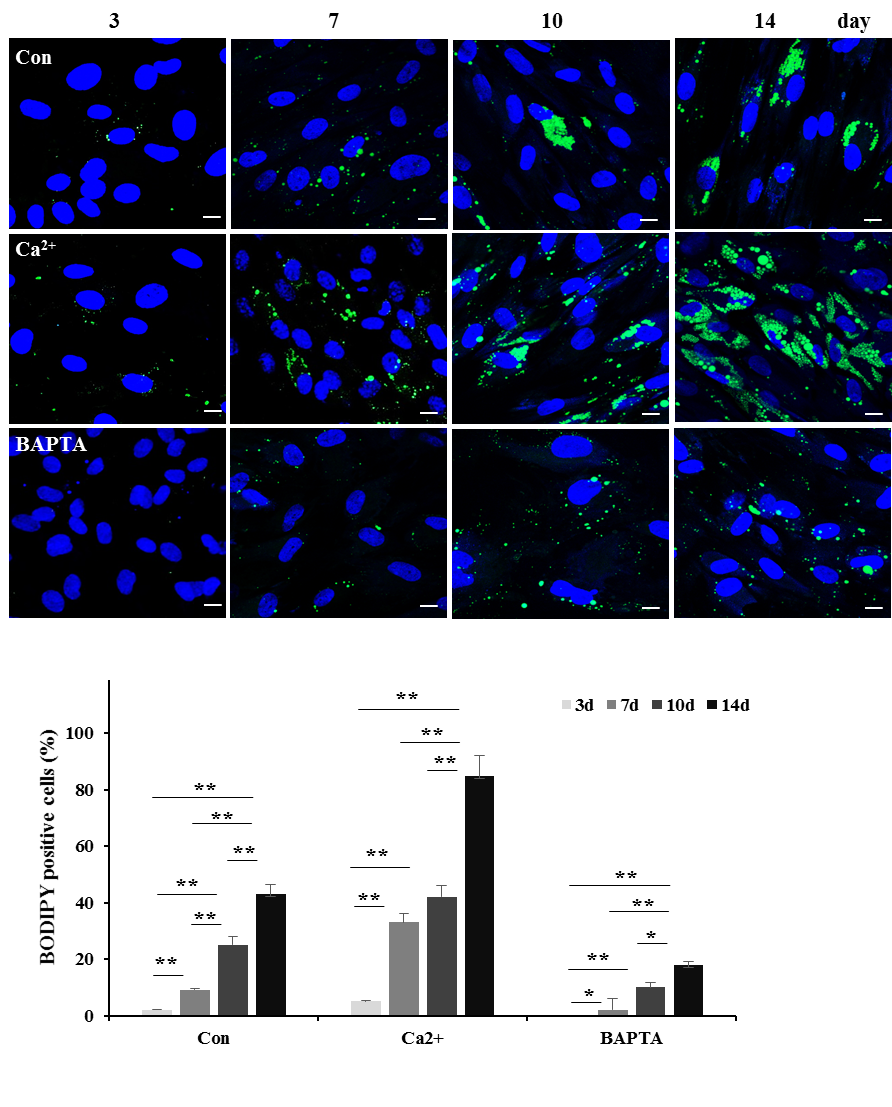


**Supplementary Figure 11. Role of Wnt5a/β-catenin signaling in adipogenic differentiation.**

(A) MSCs-H (M-H) or MSCs-L (M-L) were treated with a Wnt activator (100 ng/mL Wnt5a) or inhibitor (50 ng/mL Dkk-1) in adipogenic medium for the initial 4 days of induction. (A) At day 14 of adipogenic induction, the cells were stained with Oil red O, and activity was quantified by counting the positively stained cells (Scale bar = 50 μm, mean ± SD, n = 3, ***p* < 0.01). (B) Inhibition of β-catenin induced adipogenic differentiation in MSCs-H or MSCs-L. The cell lines were each transfected with scramble siRNA (si Con) or β-catenin siRNA (si β-cat). siRNA-mediated suppression of β-catenin expression was maintained for 15 days after transfection. Immunoblotting analysis was used to detect β-catenin signaling, with β-actin serving as a loading control. Expression levels were normalized to β-actin, with the expression levels in control the defined as 1 (right panel; mean ± SD, *n* = 3; ***p* < 0.01). (C) Cells were stained with Oil red O, and activity was quantified by counting the positively stained cells (Scale bar = 50 μm, mean ± SD, n = 3, ***p* < 0.01). ns; not significant.

**
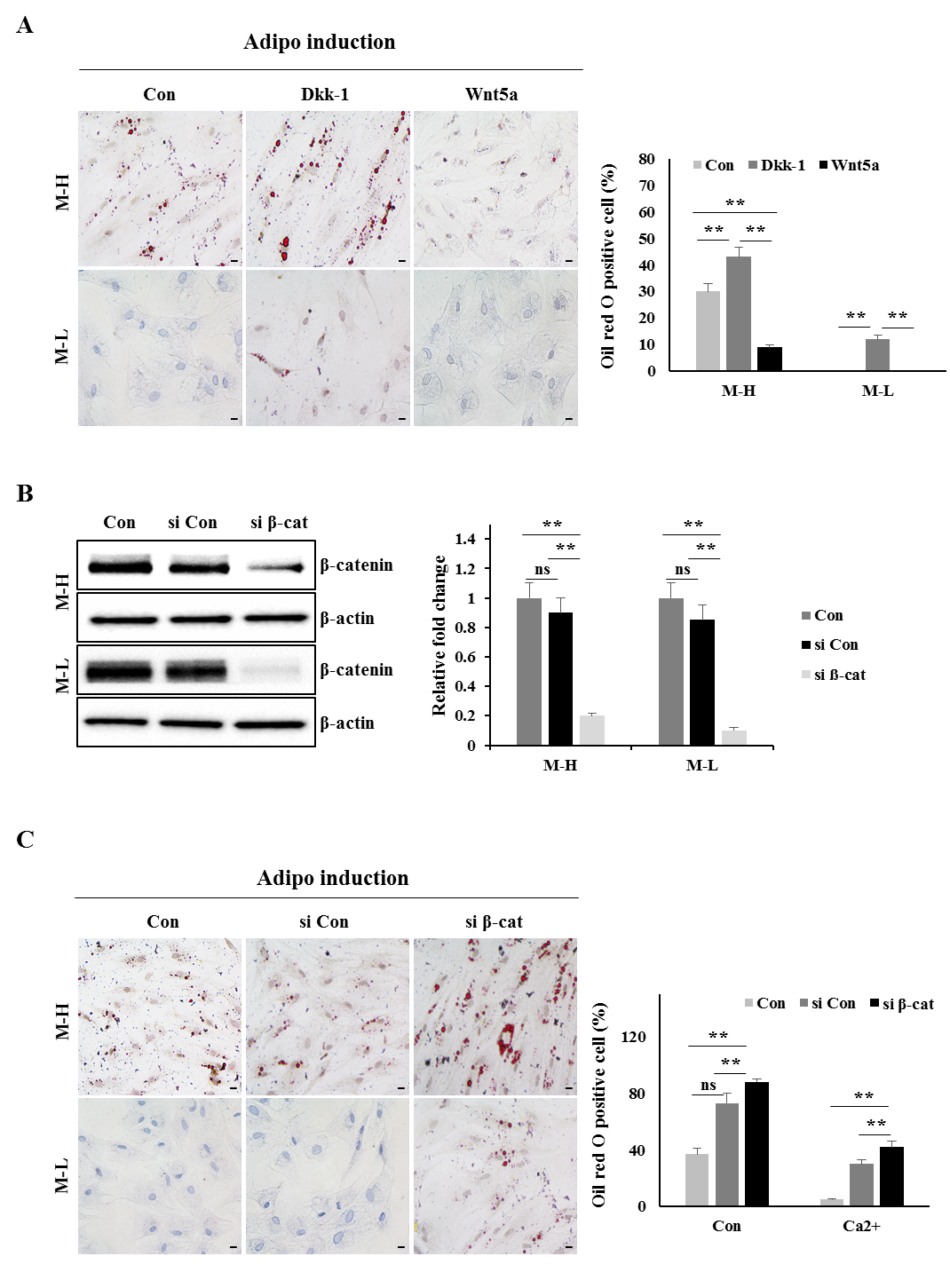
**

**Supplementary Table 1. Detailed information regarding the hUCB-MSCs used in this study.**

| **MSCs** | **Maternal age (years)** | **Cell surface marker** | | **Differentiation** |
| --- | --- | --- | --- | --- |
|  |  | **Positive** | **Negative** |  |
| #1 | 33 | Pass | Pass | Pass |
| #2 | 38 | Pass | Pass | Pass |
| #3 | 29 | Pass | Pass | Pass |
| #4 | 33 | Pass | Pass | Pass |
| #5 | 34 | Pass | Pass | Pass |
| #6 | 32 | Pass | Pass | Pass |

hUCB-MSCs were isolated from six independent donors (MSCs #1 to 6). The MSC features were analyzed by representative MSC marker expression or their capacity for differentiation (Positive: CD73, CD105, CD166 ≥ 85%; Negative: CD14, CD45, HLA-DR ≤ 1.5%; Differentiation: osteogenic, chondrogenic).

**Supplementary Table 2. Sequences of primers used for sequencing the indicated target genes.**

| **Target gene** | **Primer sequence (5′–3′)** | | **Taqman Probe** |
| --- | --- | --- | --- |
| Wnt1 | Left | cgctggaactgtcccact | 81 |
|  | Right | aacgccgtttctcgacag |  |
| Wnt3a | Left | aactgcaccaccgtccac | 64 |
|  | Right | aaggccgactccctggta |  |
| Wnt4 | Left | gcagagccctcatgaacct | 4 |
|  | Right | cacccgcatgtgtgtcag |  |
| Wnt5a | Left | attgtactgcaggtgtaccttaaaac | 48 |
|  | Right | cccccttataaatgcaactgttc |  |
| Wnt5b | Left | gcgagaagactggaatcagg | 30 |
|  | Right | cagagcagccgtgaacag |  |
| Wnt10b | Left | atgcgaatccacaacaacag | 27 |
|  | Right | tccagcatgtcttgaactgg |  |
| PPARγ | Left | gacaggaaagacaacagacaaatc | 7 |
|  | Right | ggggtgatgtgtttgaacttg |  |
| Leptin | Left | ttgtcaccaggatcaatgaca | 25 |
|  | Right | gtccaaaccggtgactttct |  |
| β-actin | Left | tccctccctggagaagcta | 64 |
|  | Right | aggaggagcaatgatcttgat |  |
| β-catenin siRNA | i | GAUCCUAGCUAUCGUUCUU | |
|  | ii | UAAUGAGGACCUAUACUUA | |
|  | iii | GCGUUUGGCUGAACCAUCA | |
|  | iv | GGUACGAGCUGCUAUGUUC | |
| Scrambled siRNA | i | UGGUUUACAUGUCGACUAA | |
|  | ii | UGGUUUACAUGUUGUGUGA | |
|  | iii | UGGUUUUUCACAUGUUUUCUG | |
|  | iv | UGGUUUACAUGUUUUUCCUA | |
